# Nuclear and Chloroplast Sequences Resolve the Enigmatic Origin of the Concord Grape

**DOI:** 10.3389/fpls.2020.00263

**Published:** 2020-03-17

**Authors:** Jun Wen, Sterling A. Herron, Xue Yang, Bin-Bin Liu, Yun-Juan Zuo, AJ Harris, Yash Kalburgi, Gabriel Johnson, Elizabeth A. Zimmer

**Affiliations:** ^1^Department of Botany, National Museum of Natural History, Smithsonian Institution, Washington, DC, United States; ^2^Donald Danforth Plant Science Center, St. Louis, MO, United States; ^3^Agriculture School, Kunming University, Kunming, China; ^4^State Key Laboratory of Systematic and Evolutionary Botany, Institute of Botany, Chinese Academy of Sciences, Beijing, China; ^5^Xishuangbanna Tropical Botanical Garden, Chinese Academy of Sciences, Mengla, China; ^6^Department of Biology, Oberlin College and Conservatory, Oberlin, OH, United States; ^7^Key Laboratory for Plant Resources Conservation and Sustainable Utilization, South China Botanical Garden, Chinese Academy of Sciences, Guangzhou, China

**Keywords:** Concord grape, grape, origin, *Vitis*, Vitaceae

## Abstract

Despite the commercial importance of the Concord grape, its origin has remained unresolved for over 150 years without a comprehensive phylogenetic analysis. In this study we aimed to reconstruct the evolutionary history of the Concord grape using sequence data from four nuclear markers (*AT103*, *GAI1*, *PHYA*, and *SQD1*), six plastid markers (*matK*, *psbA-trnH*, *petN-trnC*, *ycf1*, *trnL-F*, and *trnS-G*), and the plastid genome. We sampled extensively the *Vitis* species native to northeastern North America as well as representative species from Europe and Asia, including the commercially important *Vitis vinifera* (wine grape), a native European species with hermaphroditic flowers, and its wild progenitor, *V*. *vinifera* subsp. *sylvestris*. We also sequenced the plastid genome of one accession of the Concord grape and compared the plastid genome data to the recently published data set of *Vitis* plastomes. Phylogenetic analyses of the plastid and nuclear data using maximum likelihood and Bayesian inference support the hybrid origin of the Concord grape. The results clearly pinpoint the wine grape, *V*. *vinifera*, as the maternal donor and the fox grape, *Vitis labrusca*, which is common in northeastern North America, as the paternal donor. Moreover, we infer that the breeding history of the Concord grape must have involved the backcrossing of the F1 hybrid with the paternal parent *V*. *labrusca*. This backcrossing also explains the higher morphological similarity of the Concord grape to *V*. *labrusca* than to *V*. *vinifera*. This study provides concrete genetic evidence for the hybrid origin of a widespread *Vitis* cultivar and is, therefore, promising for similar future studies focused on resolving ambiguous origins of major crops or to create successful hybrid fruit crops.

## Introduction

The Concord grape is an economically important cultivar in the United States and Canada as a source of juice, jelly, jam, table grape, candy, and sweet wine, as well as a popular garden plant. It is a hardy and productive vine that bears hermaphroditic flowers and large blue-black berries. However, the origin of the Concord grape has been ambiguous since its introduction by Ephraim Wales Bull in the town of Concord, MA, United States in the 1840’s. Bull labored for 6 years on his grape plants in order to develop the perfect, cold-hardy crop with hermaphroditic flowers (Schofield, 1988). Many early attempts to grow European *Vitis vinifera* cultivars failed in North America due to diseases and pests such as phylloxera as well as climatic reasons (Gerrath et al., 2015). The introduction of the Concord grape, which was well adapted to conditions in the eastern United States, was revolutionary to grape cultivation (Miller, 1954). Many workers believe that the Concord grape was derived from selection from the native local fox grape *Vitis labrusca* that Bull planted from seeds (Munson, 1909; Galet, 1979; Schofield, 1988). Schofield (1988) cited that Bull told Liberty Hyde Bailey decades later that “boys brought up from the Concord River some wild grapes and scattered them about the place.” Bull is then reported to have used the seeds of these grapes to produce the Concord variety (Schofield, 1988). However, a pure selection scenario may be less likely considering the relatively short time frame in which the Concord grape was developed. Others argue that it is a hybrid of two or more grape species (Munson, 1909; Bailey, 1934). Munson (1909) hypothesized that the Concord grape was primarily derived from *V*. *labrusca* L. but might contain a trace of *V*. *vulpina* L. (=*V*itis *riparia* Michx. sensu Moore and Wen, 2016, not sensu Munson as *V*. *riparia* was misidentified as *V*. *vulpina* by Munson). *V*. *riparia* is a common native species near Concord and throughout the northern part of North America. However, the wine grape *V*. *vinifera* L. was also regarded as a potential parent due to the Concord grape having hermaphroditic flowers, a trait only found in *V*. *vinifera* (Munson, 1909; Schofield, 1988), even though the Concord grape is highly similar to the fox grape *V*. *labrusca* in many other morphological characters.

The grape genus, *Vitis* L., is one of 16 genera in the grape family Vitaceae (Wen, 2007; Wen et al., 2018b). *Vitis* includes about 70 species primarily distributed in Asia, and North to Central America, and has one species in Europe (Chen et al., 2007; Wen, 2007; Moore and Wen, 2016; Wen et al., 2018a). *Vitis* has been classified into two subgenera: subgenus *Vitis* (c. 68 spp.) and subgenus *Muscadinia* (Planch.) Rehder (2 spp.) (Brizicky, 1965; Moore, 1991; Wen, 2007; Wen et al., 2018b). The chromosome number for *Vitis* subgenus *Vitis* is 2*n* = 38, with no polyploidy having been reported for the genus (Wen, 2007). Several recent studies have shed new light on the phylogeny of *Vitis* (Aradhya et al., 2008, 2013; Tröndle et al., 2010; Péros et al., 2011; Zecca et al., 2012; Miller et al., 2013; Wan et al., 2013; Liu et al., 2016; Klein et al., 2018; Ma et al., 2018a, b; Wen et al., 2018a). Notably, there is incongruence among studies concerning the monophyly of the taxa of *Vitis* subgenus *Vitis* in North America (cf. Wan et al., 2013; Liu et al., 2016; Ma et al., 2018a; Wen et al., 2018a). Nevertheless, several studies have resolved *Vitis* subgenus *Vitis* into two main clades corresponding to their geographic distributional areas in Eurasia and North America (e.g., Zecca et al., 2012; Klein et al., 2018; Ma et al., 2018b; Wen et al., 2018a).

The goal of this study was to clarify the genetic donor(s) of the Concord grape using a broad sampling scheme of the putative relatives from North America, especially from northeastern North America. To achieve our goal, we performed phylogenetic analyses on plastid (*matK*, *trnL-F*, *petN-trnC*, *trnH-psbA*, *trnS-G*, and *ycf1*) and nuclear (*GAI1*, *AT103*, *PHYA*, and *SQD1*) sequence data from the sampled *Vitis* species. The markers were selected based on our success in using them in prior phylogenetic studies of plant species (Ren et al., 2011; Zimmer and Wen, 2012; Zhao et al., 2016, 2018; Hearn et al., 2018; Lu et al., 2018). Even though *GAI1* (the grapevine derived *GA INSENSITIVE* or *GAI*-like gene), the phytochrome genes (e.g., *PHYA*), and the sulfoquinovosyldiacylglycerol 1 (or *SQD1*) gene may have significant functions (Li et al., 2008; Zimmer and Wen, 2012), our purpose of utilizing these markers in this study is as nuclear phylogenetic markers as discussed in detail in Zimmer and Wen (2012). We also sequenced the plastid genome of one accession of the Concord grape and analyzed it alongside the recently published data set of plastomes of *Vitis* (Wen et al., 2018a). Our work has implications not only for understanding the cultivation history of this species, but also for future breeding efforts of grape cultivars.

## Materials and Methods

### Taxon Sampling

To achieve a comprehensive sampling of the species which Mr. Bull may have selected for his breeding of the Concord grape, we collected specimens of *Vitis* from North America emphasizing the northeastern United States, including the following species: *Vitis acerifolia* Raf., *V. aestivalis* Michx., *V. arizonica* Engelm., *V. cinerea* (Engelm.) Millardet [var. *baileyana* (Munson) Comeaux, var. *cinerea* and var. *floridana* Munson], *V*. *labrusca* L., *V*. *mustangensis* Buckley, *V*. *riparia* Michx., *V*. *rotundifolia* Michx., *V*. *vulpina* L. and the cultivated wine grape *V*. *vinifera* L. (Moore and Wen, 2016). Five cultivars of *V*. *vinifera* were included: Pinot Noir, Reichensteiner, Riesling, Syrian, and Thompson Seedless, to represent the gene pool of the wine grape as a potential parental species, not necessarily to genotype the exact cultivar that Mr. Bull might have used to breed the Concord grape. The original Concord grape *V*. *labruscana* L. H. Bailey grown by Ephraim Bull was sampled (*Wen 12570*) from the Grape Cottage in Concord, Massachusetts, as well as a cutting from the original Concord grape growing at Welch’s Foods Inc., in Concord, MA (*Wen 12568*). Furthermore, we included representative taxa of *Vitis* from Eurasia, *V*. *flexuosa* Thunb. (var. *flexuosa* and var. *parvifolia* (Roxb.) Gagnep.), *V*. *lanata* Roxb. ex Wall., *V*. *thunbergii* Siebold and Zucc. and *V*. *vinifera* subsp. *sylvestris* (Gmelin) Hegi (Supplementary Table S1). The overall sampling was designed to include all potential species of *Vitis* that Mr. Bull may have had access to in Concord, Massachusetts at that time. The most likely species that he may have possessed were the native species of *Vitis* from northeastern North America and the various cultivars of *V*. *vinifera*. Therefore, we included five cultivars of the wine grape, and all native species from northeastern North America, as well as a few other Eurasian taxa sampled to ensure that the phylogenetic diversity of the genus was also represented.

We also compared the plastid genome sequence of the Concord grape with a large published data set of North American *Vitis* plastid genome sequences with two accessions from Europe and West Asia (*V*. *vinifera* ssp. *vinifera* and *V*. *vinifera* ssp. *sylvestris*), and nine representative species from eastern Asia to cover the morphological diversity of subgenus *Vitis* (Wen et al., 2018a).

### DNA Extraction, Amplification, and Sanger Sequencing

We selected ten DNA markers for resolving phylogenetic relationships and parentage in this study: six plastid (*matK*, *psbA-trnH*, *petN-trnC*, *ycf1*, *trnL-F*, and *trnS-G*) and four nuclear (*AT103*, *SQD1*, *PHYA*, and *GAI1*). Throughout, we follow the accepted nomenclature for these genes according to UniProt^1^ (The UniProt Consortium., 2017). *AT103* (or *CRD1*) is involved in chlorophyll biosynthesis (e.g., Gene Ontogeny term, GO:0015995; Bang et al., 2008), while *SQD1* has a role in synthesis of thylakoid membrane structures (e.g., GO:0046507; Sanda et al., 2001), and *PHYA* is a well-known member of the phytochrome family involved in photoperiodism and other photo-regulated pathways (e.g., GO:0010161, GO:0031516; Kim et al., 2002; Yang et al., 2009). *GAI1* is known especially from *Vitis* and its close relatives, but is likley a transcription factor in the gibberellin (GA) signaling pathway related to *RGA* in *Arabidopsis thaliana* (L.) Heynh. (e.g., GO:0009740; Silverstone et al., 1998) according to a protein BLAST (Altschul et al., 1990) search using Uniprot accession Q8S4W7 (*V*. *vinifera* L.) performed on the NCBI webserver (Madden et al., 1996). Each of these genes are essential for plant growth and almost assuredly play roles in responses to environmental stimuli that merit investigation outside of the context of this molecular phylogenetic study, in which we use them to resolve evolutionary relationships. For the purposes here, it is noteworthy that all four nuclear genes are ubiquitous in vascular plants and yield phylogenies believed to be consistent with the vascular plant tree of life (Vandenbussche et al., 2007; Mathews, 2010; Chen et al., 2017).

We extracted DNA from leaf tissue samples, dried in silica gel, using the DNeasy Plant Mini Kit (QIAGEN, Valencia, CA, United States) following a modification of the manufacturer’s protocol. For each sample, six separate lysate solutions were prepared and processed through a single QIAShredder column and DNeasy column. We amplied the ten selected markers using standard polymerase chain reactions (PCR). Primer information for the six plastid markers can be found in Taberlet et al. (1991), Soejima and Wen (2006), Ren et al. (2011), and Lu et al. (2013, 2018); and that of the nuclear markers can be located in Wen et al. (2007), Li et al. (2008), Nie et al. (2012), Liu et al. (2016), and Lu et al. (2018). The PCRs were carried out in 25 μL that contained 1.5 mM MgCl_2_, 0.2 mM of each dNTP, 0.4 mM of each primer, 1.0 U of Taq polymerase (Bioline, Aberdeen, United Kingdom), and 10–50 ng (2.5 μL) template DNAs. The amplification reactions for all ten genes were run with the following PCR program: (1) a denaturation step at 94°C for 5 min, (2) 35 cycles with a denaturing step at 94°C for 45 s, an annealing step at 50°C for 45 s, and an extension step at 72°C for 90 s, and (3) a final extension at 72°C for 10 min, using a BioRad T100 thermal cycler (Bio-Rad Laboratories, Inc., Hercules, CA, United States). The PCR products were purified using the ExoSAP-IT enzyme (cat. #78201, USB Corporation, Cleveland, OH, United States) based on the manufacturer’s protocol. Sequencing primers were the same as amplification primers, and fluorescently labeled Sanger fragments were generated using BigDye^TM^ Terminator v3.1 cycle sequencing kit (cat. #4337455, Thermo Fisher Scientific, Inc., Waltham, MA, United States) at 1/4 of the manufacturer’s suggested concentration. The resulting products were read on an ABI 3730xl automated capillary sequencer (Applied Biosystems, Foster City, CA, United States), following the manufacturer’s protocols, at the Laboratories of Analytical Biology at the National Museum of Natural History, the Smithsonian Institution (Washington, DC, United States).

### Plastid Genome Sequencing and Assembly

We sequenced the plastid genome for one accession of the Concord grape (*Wen 12529*) using the genome skimming approach (Zhang et al., 2015). The genomic library was constructed with the NEBNext Ultra II library prep kit for Illumina (New England Biolabs, Ipswich, MA, United States). Paired-end reads (2 × 150 bp) were produced using an Illumina NextSeq 500 Sequencing System at the Genomic Sequencing and Analysis Facility (GSAF) at the University of Texas, Austin. The raw reads were filtered and trimmed to remove adapters and lower quality bases at the end using Trimmomatic version 0.32 (Bolger et al., 2014) with default settings. The trimmed paired-end reads were used to assemble the plastid genome with NOVOPlasty 3.2 (Dierckxsens et al., 2016). The plastid genome sequence of the Concord grape was then analyzed phylogenetically with sequences of *Vitis* from Wen et al. (2018b).

### Sequence Alignment and Phylogenetic Analyses

We performed multiple sequence alignments of the datasets in MAFFT (Katoh et al., 2002; Katoh and Toh, 2008), followed by manual adjustment within Geneious 10.2.4 (Kearse et al., 2012).

The best fit partitioning schemes and nucleotide substitution models for the data sets (whole plastome, combined plastid regions, *AT103*, *GAI1*, *PHYA*, *SQD1*, and combined nuclear sequences) were estimated using PartitionFinder2 (Lanfear et al., 2016). Under the corrected Akaike information criterion (AICc) and linked branch lengths, PartitionFinder2 was performed with the greedy (Lanfear et al., 2012) and rcluster (Lanfear et al., 2014) algorithm options for these three datasets, with prior defined data blocks by codon positions of each protein-coding gene and all models. The partitioning schemes and evolutionary model for each subset were used for the downstream maximum likelihood (ML, Stamatakis, 2006, 2014) and Bayesian Inference (BI, Rannala and Yang, 1996; Mau et al., 1999) analyses. The ML trees were inferred by IQ-TREE v.1.6.9 (Nguyen et al., 2015) with 1000 bootstrap replicates using UFBoot2 (Hoang et al., 2017) and the collapsing near zero branches option. The BI was performed with MrBayes 3.2.7 (Ronquist et al., 2012). The Markov chain Monte Carlo (MCMC) analyses were run for 10,000,000 generations. Trees were sampled at every 1,000 generations with the first 25% discarded as burn-in. The remaining trees were used to build a 50% majority-rule consensus tree. The stationarity was considered to be reached when the average standard deviation of split frequencies remained below 0.01. The ML and BI trees were visualized with Tree View using Geneious Prime (Kearse et al., 2012).

## Results

**FIGURE 1:**
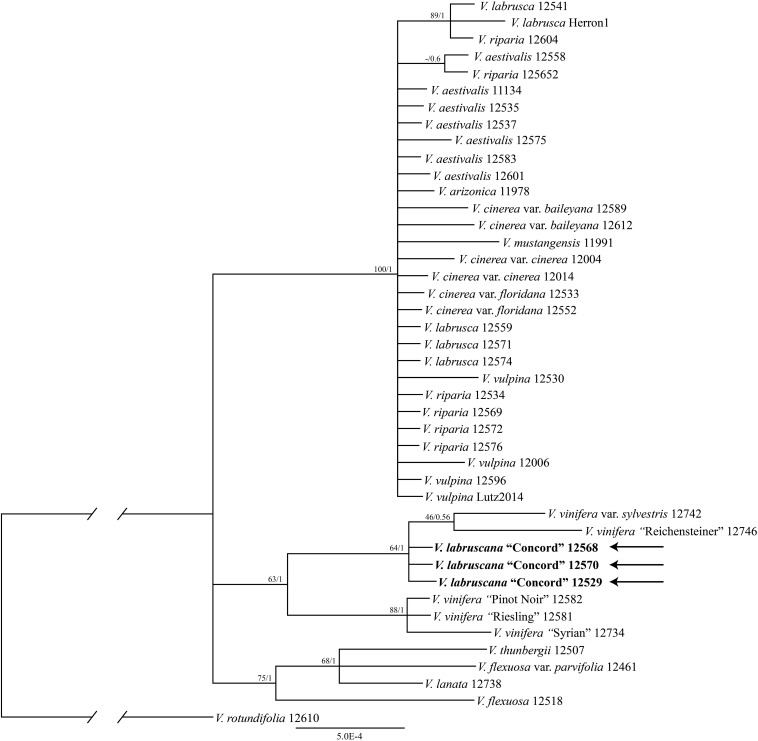
Phylogenetic relationships of the Concord grape and its close allies in *Vitis* using combined chloroplast DNA marker sequences and Bayesian inference. Numbers associated with the branches are ML bootstrap support and Bayesian posterior probability values.

**FIGURE 2:**
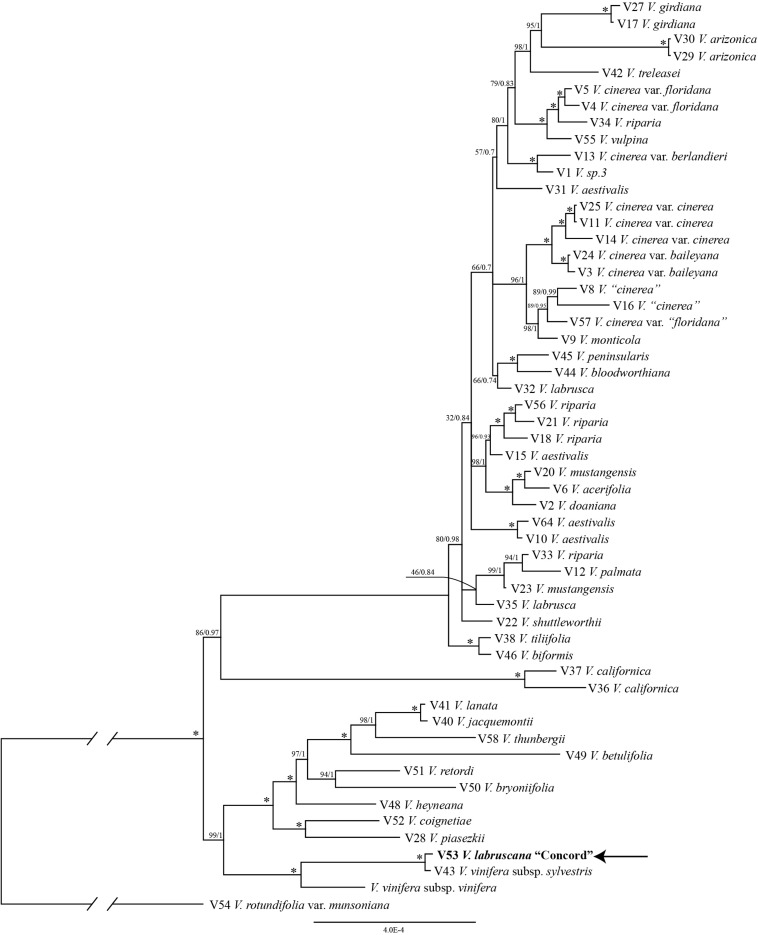
Phylogenetic relationships of the Concord grape and its close allies in *Vitis* using complete chloroplast DNA genome sequences and Bayesian inference. Numbers associated with the branches are ML bootstrap support and Bayesian posterior probability values, and asterisks (^∗^) indicate bootstrap support/posterior probability of 100/1.00.

The trees from the combined Sanger plastid data placed the samples of the Concord grapes nested within the Eurasian *V*. *vinifera* clade (Figure 1). The clade of Concord grapes plus the Eurasian *V*. *vinifera* (both subsp. *vinifera* and subsp. *sylvestris*) had bootstrap support of 63% and a Bayesian posterior probability of 1.00 (Figure 1). The maximum likelihood analysis and Bayesian inference of the plastid genome data of *Vitis* generated an identical topology, which placed the Concord grape (*Wen 12529*, chloroplast genome GenBank accession number MN577933) as nested within *V*. *vinifera* (sister to *V*. *vinifera* subsp. *sylvestris*) (Figure 2). Of great interest, there are several significant insertions and deletions in the plastid DNA, such as a 5-bp insertion in the *trnL-F* region and a 54-bp deletion in the *petN-trnC* intergenic spacer that were only shared between the Concord grape and *V*. *vinifera*.

Each of the nuclear gene regions had very few informative sites. Separate analyses of the four nuclear gene regions did not provide much phylogenetic resolution (Supplementary Figures S1–S4) except that the *SQD1* tree supported a clade of *V*. *labrusca* and the Concord grape (Supplementary Figure S1). Furthermore, the *AT103* data showed that the Concord grape had two recombinational sites between *V*. *labrusca* and *V*. *vinifera*, supporting the hybrid status of the Concord grape.

**FIGURE 3:**
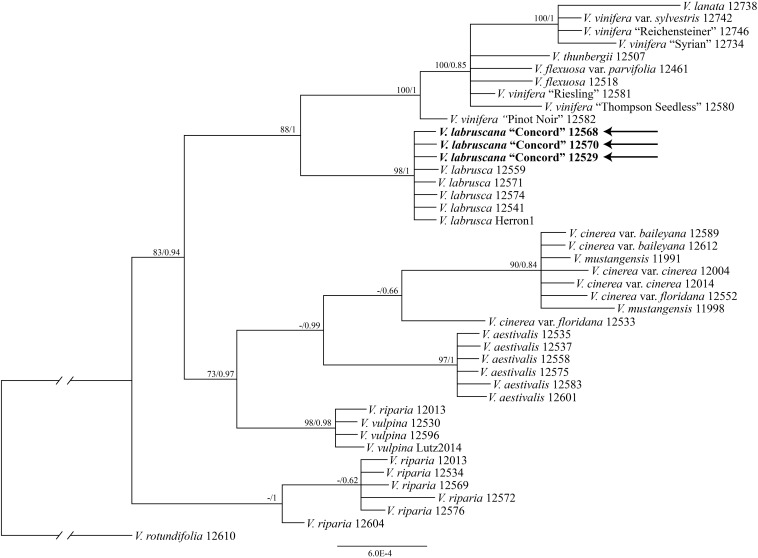
Phylogenetic relationships of the Concord grape and its close allies using combined nuclear sequences and Bayesian inference. Numbers associated with the branches are ML bootstrap support and Bayesian posterior probability values.

The tree of the combined nuclear data (*AT103*, *GAI1*, *PHYA*, and *SQD1*) strongly supported that the Concord grape samples formed a clade with multiple samples of the fox grape *V*. *labrusca* from eastern North America (bootstrap support 98%, PP 1.00; Figure 3).

The plastid and nuclear trees clearly showed topological incongruence concerning the position of the Concord grape (cf. Figures 1–3). Furthermore, all samples of *V*. *riparia* formed a clade in the nuclear tree, but they did not constitute a monophyletic group in the plastid tree (Figure 2). A similar pattern of discordance is observed in *V*. *aestivalis*, although our sampling covered only *V*. *aestivalis* var. *aestivalis* in this study. A pattern of nuclear monophyly and plastid non-monophyly is also seen in *V*. *vulpina* (Figures 2, 3). *Vitis cinerea* showed a complex pattern such that the three varieties, var. *baileyana*, var. *cinerea* and var. *floridana*, did not form a clade.

## Discussion

### Hybrid Origin of the Concord Grape

The plastid results (the combined 6-marker data as well as the complete plastid genome data; Figures 1, 2) show that the Concord grape forms a clade with the Eurasian *V*. *vinifera*. As plastid DNA is maternally inherited, the close alliance of the Concord grape accessions with *V*. *vinifera* (including two subspecies) suggests that *V*. *vinifera* was the maternal parent from which the Concord grape was derived. With their shared hermaphroditic flowers, a relationship between the Concord grape and *V*. *vinifera* had long been suspected (Munson, 1909; Schofield, 1988). Within *Vitis*, hermaphroditic flowers are only predominantly found in *V*. *vinifera* ssp. *vinifera* (Chen et al., 2007; Moore and Wen, 2016; Gerrath et al., 2017). Nevertheless, it has also been proposed that the Concord grape may have been developed from the fox grape *V*. *labrusca* alone by repeated rounds of selection (Galet, 1979; Gerrath et al., 2015). The chloroplast topology clearly refutes the selection hypothesis, and instead shows the genetic relationship of the Concord grape with the wine grape *V*. *vinifera*. The Concord grape is best interpreted as a hybrid that involved *V*. *vinifera* as the maternal parent.

**FIGURE 4:**
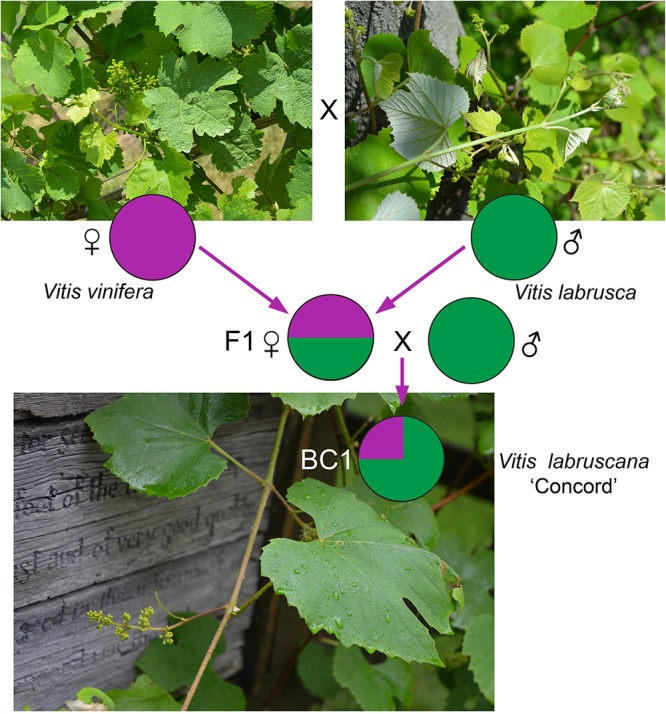
A model for the Concord grape origin based on chloroplast and nuclear DNA evidence. BC1 represents the backcrossed progeny of the F1 hybrid toward one of the parental species, *Vitis labrusca*.

The strong similarity between the nuclear sequences of the Concord grape and the fox grape *V*. *labrusca* suggests the latter as the paternal parent of the Concord grape. The distinct nuclear sequence similarities between *V*. *labrusca* and the Concord grape, as well as their morphological similarities, indicate that the F1 hybrid was backcrossed with *V*. *labrusca* in the development of the Concord grape by Bull (Figure 4). Morphologically the Concord grape possesses continuous tendrils and a whitish to rusty tomentum on the adaxial surface of the leaf blade, similar to the fox grape *V*. *labrusca*.

The comparative genomics study by Sawler et al. (2013), using SNP data, reported that the Concord grape contains c. 30% of the *V*. *vinifera* genome. Based on this evidence, the backcrossing likely occurred just once (Figure 4); in this scenario, the Concord grape would contain c. 75% of the *V*. *labrusca* nuclear genome and c. 25% of the *V*. *vinifera* nuclear genome. *V*. *labruscana* L. H. Bailey has been commonly used to designate American grape cultivars that have a *V*. *labrusca* parentage (Bailey, 1934; Moore and Wen, 2016).

Ephraim Wales Bull passed away in 1895 without profiting financially from the great Concord grape that he cleverly created. The epitaph on his tombstone reads: “He Sowed, Others Reaped” (Schofield, 1988). We hope our deciphering of the enigmatic origin of the Concord grape will help bring due honor to such an ingenious plant breeder for his labor and legendary contribution to the American grape culture!

### A Glimpse Into the Discordance Between Chloroplast and Nuclear Data in *Vitis*

It is worth noting that our plastid and nuclear DNA trees for *Vitis* showed some topological discordances (c.f. Figures 1–3). Of particular interests, a pattern of nuclear monophyly and cpDNA non-monophyly was seen in *V*. *aestivalis*, *V*. *riparia* and *V*. *vulpina* (Figures 2, 3). *Vitis cinerea* seems to present a complex pattern both in the nuclear and plastid DNA trees. Currently five varieties are recognized within *V*. *cinerea* (Moore and Wen, 2016), and our sampling included only var. *baileyana*, var. *cinerea* and var. *floridana*. The three varieties did not form a clade, and their taxonomic status needs to be reassessed [also see section “Discussion” in Wen et al. (2018b)].

Many mechanisms may contribute to topological incongruence, especially lineage sorting, hybridization, and introgression (Soltis and Kuzoff, 1995; Hipp et al., 2004; Yi et al., 2015). Hybridization among North American *Vitis* species has long been discussed (Bailey, 1897, 1934; Munson, 1909; Comeaux et al., 1987; Moore, 1991; Aradhya et al., 2013; Wan et al., 2013; Moore and Wen, 2016; Wen et al., 2018a); and introgression has recently been proposed as an important driver for North American *Vitis* diversification (Nie et al., 2019). Thus, our preliminary data on the incongruence between the nuclear and plastid markers are consistent with the hypothesis of extensive reticulate evolution in North American *Vitis*. The nuclear gene tree (Figure 3) suggests that widespread species such as *V*. *aestivalis*, *V*. *riparia* and *V*. *vulpina* may have served as pollen donors in multiple hybridization events within *Vitis* (cf. Figures 1–3). Much work remains to be done concerning the patterns of hybridization and introgression and their potential impact on North American *Vitis* taxonomy, conservation and utilization (Moore and Wen, 2016; Wen et al., 2018a). We will explore incongruence among these data and its likely mechanisms using a broader taxon sampling scheme and additional genes from both the nuclear and plastid genomes in the near future using the target enrichment approach (Weitemier et al., 2014; Wanke et al., 2017; Kleinkopf et al., 2019; Li et al., 2019; Nie et al., 2019).

## Data Availability Statement

The sequencing data generated in this study has been deposited in the GenBank of NCBI and can be found using accession numbers MN702013–MN702384.

## Author Contributions

JW and EZ conceived and oversaw the study. SH, YK, AH, XY, B-BL, Y-JZ, and GJ performed experiments and implemented the data analyses. JW collected the specimens. JW, SH, and EZ wrote the manuscript. All authors read the manuscript and approved the final version.

## Conflict of Interest

The authors declare that the research was conducted in the absence of any commercial or financial relationships that could be construed as a potential conflict of interest.

## Footnotes

1 https://www.uniprot.org/
