# Supplementary figures and images for: Nuclear and Chloroplast Sequences Resolve the Enigmatic Origin of the Concord Grape
Source: Front Plant Sci. 2020 Mar 17;11:263. doi: 10.3389/fpls.2020.00263 (PMC7092692; doi:10.3389/fpls.2020.00263)

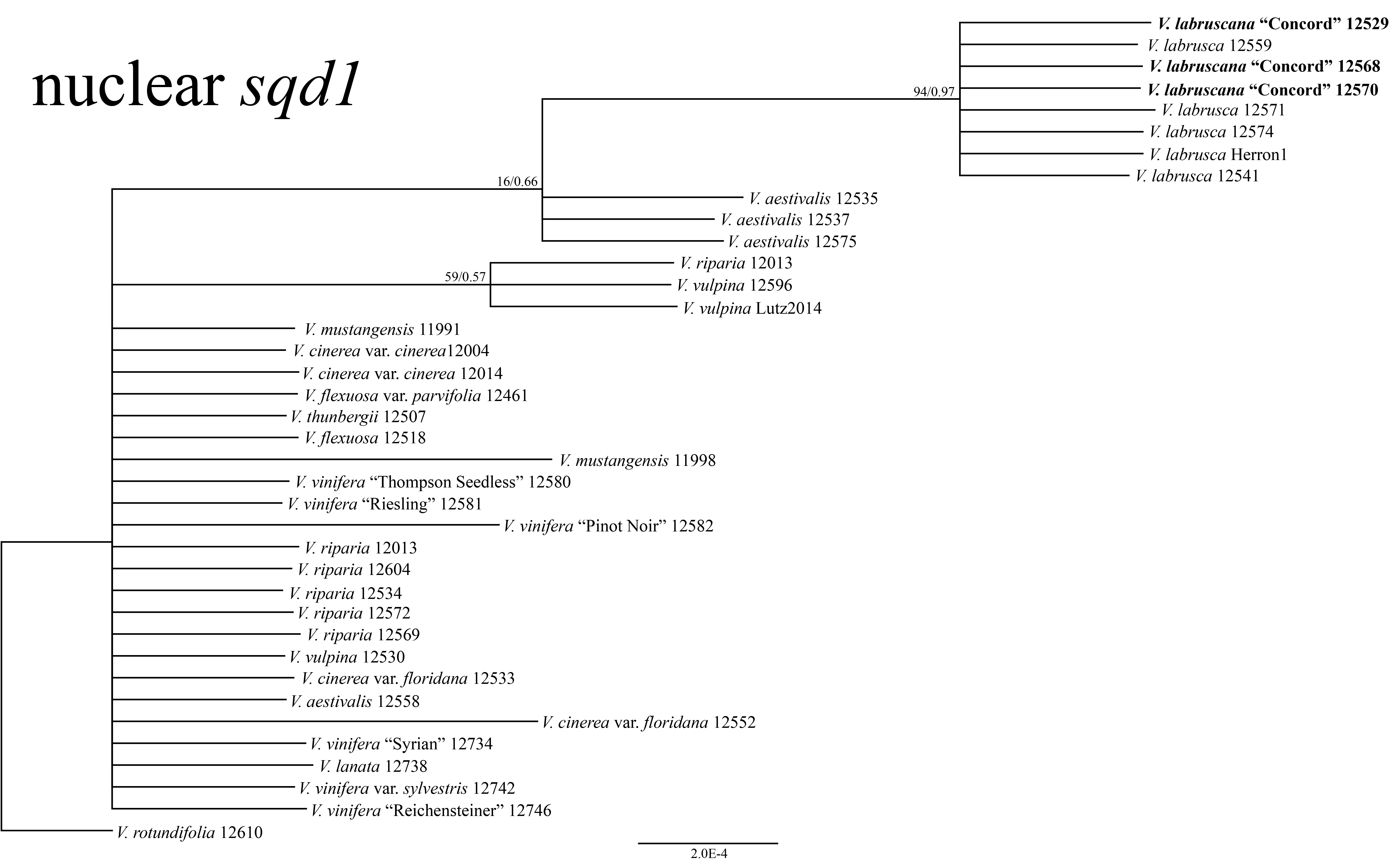

Supplement: Supplementary file 1 [file Image_1.JPEG]

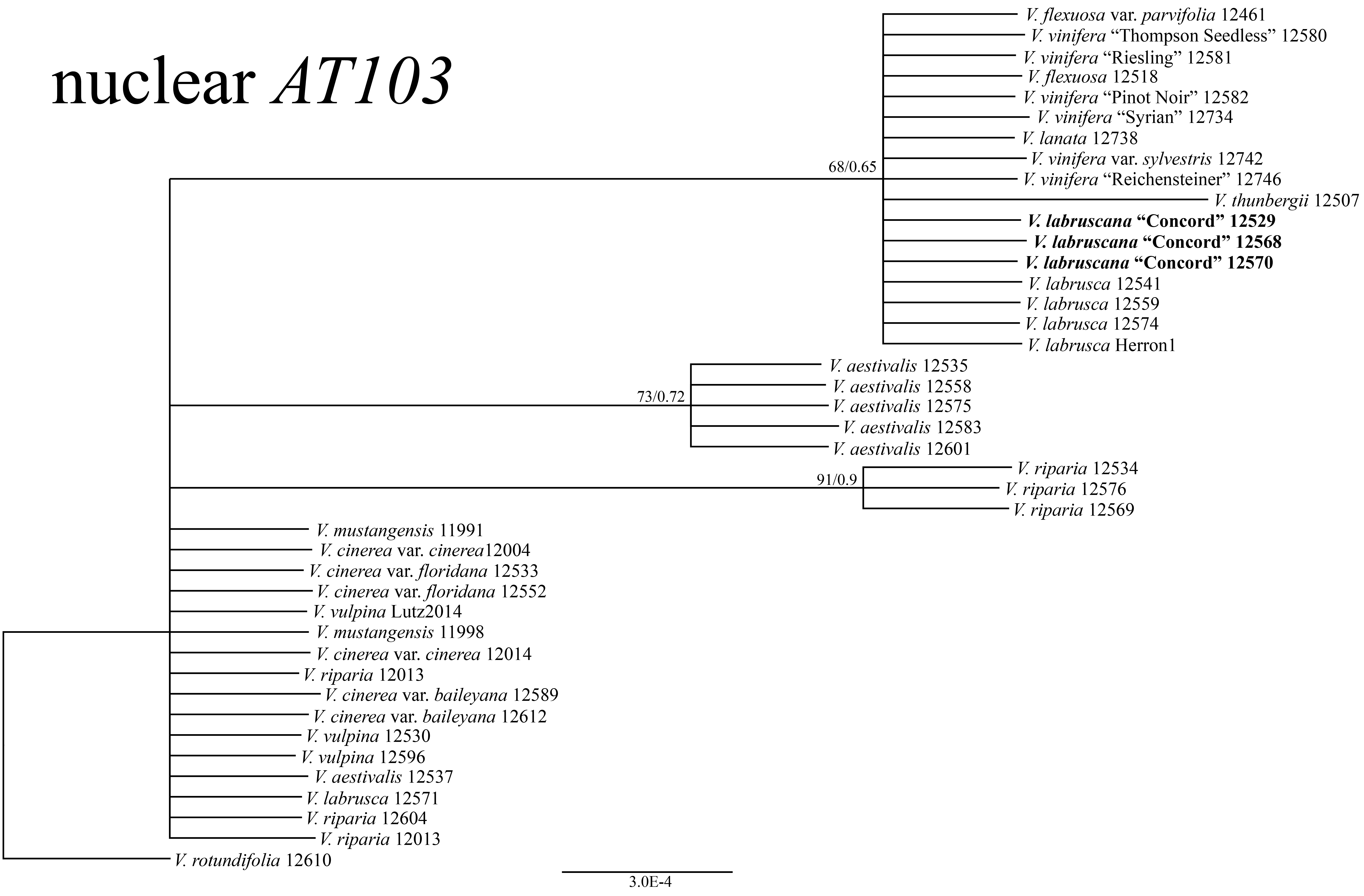

Supplement: Supplementary file 2 [file Image_2.JPEG]

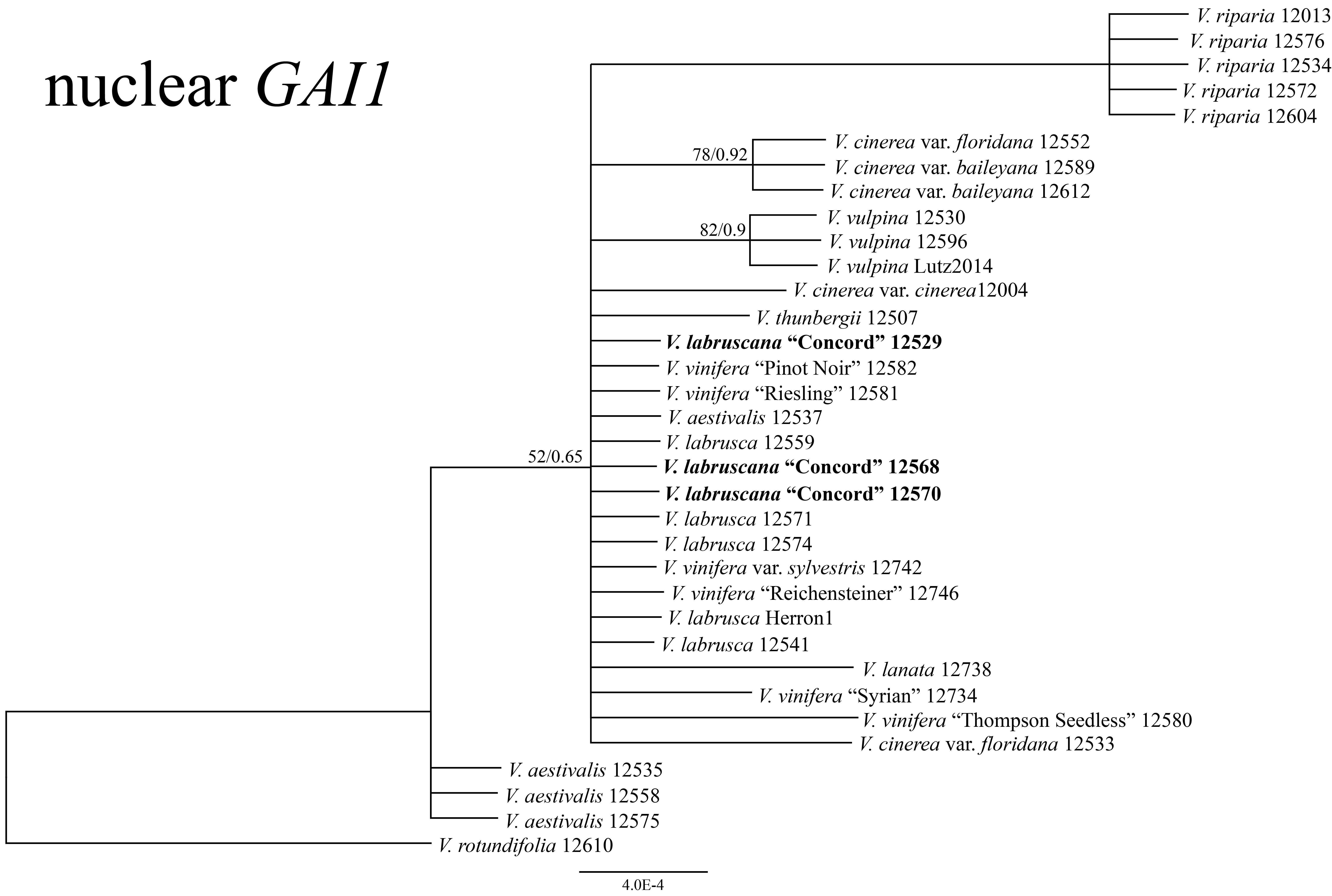

Supplement: Supplementary file 3 [file Image_3.JPEG]

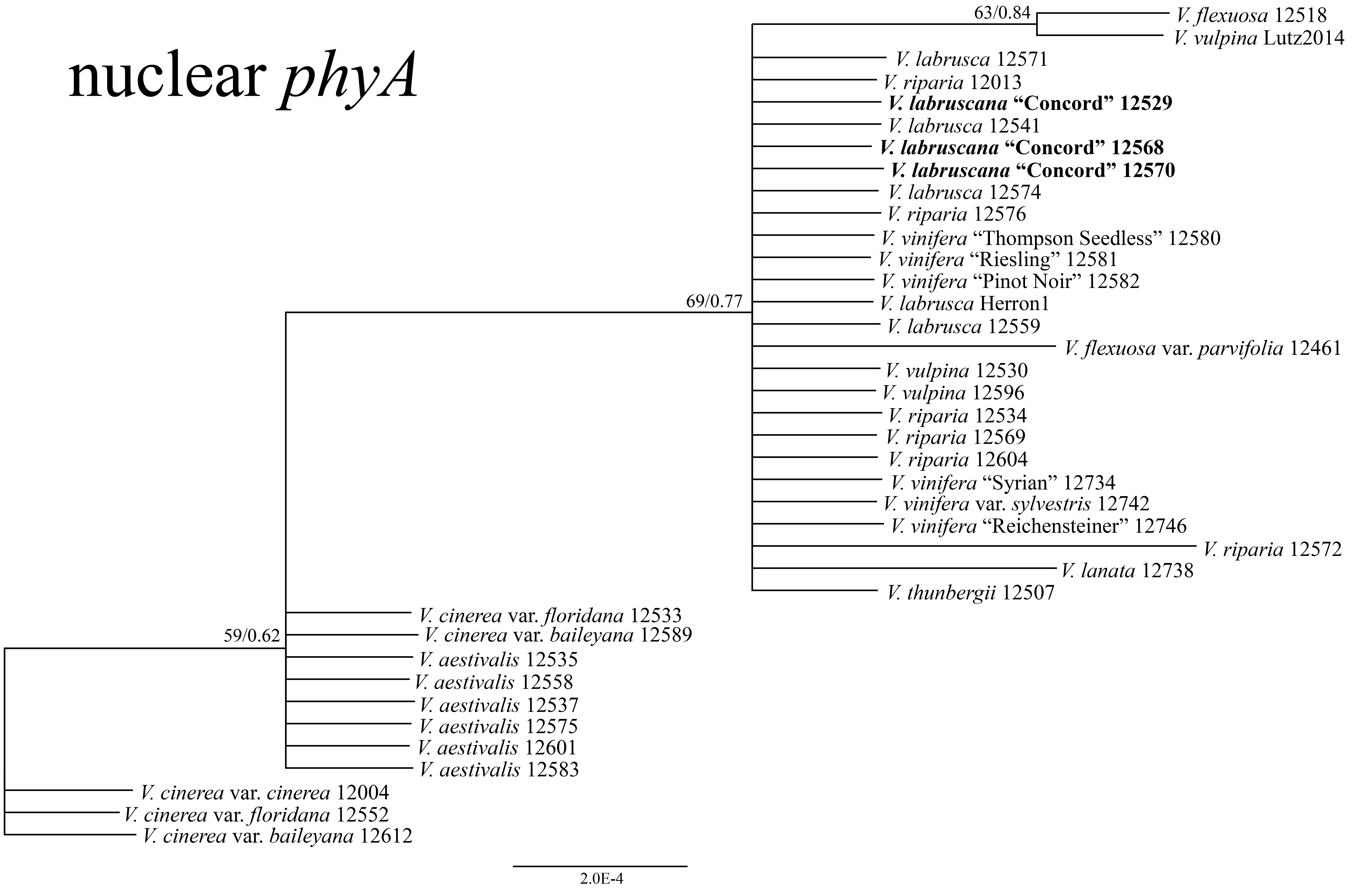

Supplement: Supplementary file 4 [file Image_4.JPEG]
