# Supplementary material for: Nuclear and Chloroplast Sequences Resolve the Enigmatic Origin of the Concord Grape
Source: Front Plant Sci. 2020 Mar 17;11:263. doi: 10.3389/fpls.2020.00263 (PMC7092692; doi:10.3389/fpls.2020.00263)
Supplement: Supplementary file 5 [file Table_1.docx]

Table S1. Information and accessions of Concord grape and the close relatives of *Vitis* included in the study. The voucher specimens are deposited at the United States National Herbarium (US).

| Taxon | Collection | Locality | GenBank Accessions (chloroplast regions) | | | | | | GenBank Accessions (nuclear sequences) | | | |
| --- | --- | --- | --- | --- | --- | --- | --- | --- | --- | --- | --- | --- |
|  |  |  | *matK* | *psbA-trnH* | *trnC-petN* | *trnL-trnF* | *trnS-trnG* | *ycf1* | *AT103* | *GAI1* | *PHYA* | *SQD1* |
| *V. aestivalis* | *Wen 11134* | South Carolina, Charleston Co. | MN702193 | MN702235 | MN702015 | - | MN702082 | - | - | - | - | - |
| *V. aestivalis* | *Wen 12535* | Maryland, Montgomery Co. | MN702206 | - | MN702028 | - | MN702093 | MN702277 | MN702320 | MN702128 | MN702164 | MN702362 |
| *V. aestivalis* | *Wen 12537* | Maryland, Fredrick Co. | MN702207 | MN702239 | MN702029 | - | MN702094 | MN702278 | MN702321 | MN702129 | MN702165 | MN702363 |
| *V. aestivalis* | *Wen 12558* | New Jersey, Trenton | MN702210 | MN702242 | MN702032 | MN702057 | MN702097 | MN702281 | MN702324 | MN702132 | MN702168 | MN702366 |
| *V. aestivalis* | *Wen 12575* | New York, Orange Co. | MN702218 | MN702250 | MN702040 | MN702065 | MN702105 | MN702289 | MN702331 | MN702139 | MN702176 | MN702374 |
| *V. aestivalis* | *Wen 12583* | Virginia, Rockbridge Co. | MN702222 | MN702254 | MN702044 | MN702069 | MN702109 | MN702293 | MN702336 | - | MN702181 | - |
| *V. aestivalis* | *Wen 12601* | Virginia, Dickenson Co. | MN702225 | MN702257 | MN702047 | - | - | MN702296 | MN702339 | - | MN702184 | - |
| *V. arizonica* | *Wen 11978* | Texas, Jeff Davis Co. | MN702194 | MN702236 | MN702016 | - | - | - | - | - | - | - |
| *V. cinerea* var. *baileyana* | *Wen 12589* | Virginia, Bland Co. | MN702223 | MN702255 | MN702045 | MN702070 | - | MN702294 | MN702337 | MN702144 | MN702182 | - |
| *V. cinerea* var. *baileyana* | *Wen 12612* | Virginia, Grayson Co. | MN702228 | MN702260 | MN702050 | MN702074 | MN702113 | MN702299 | MN702342 | MN702148 | MN702186 | - |
| *V. cinerea* var. *cinerea* | *Wen 12004* | Texas, Montgomery Co. | MN702196 | - | MN702018 | - | MN702084 | MN702269 | MN702309 | MN702121 | MN702155 | MN702351 |
| *V. cinerea* var. *cinerea* | *Wen 12014* | Mississippi, Scott Co. | MN702198 | MN702238 | MN702020 | - | MN702086 | - | MN702311 | - | - | MN702353 |
| *V. cinerea* var. *floridana* | *Wen 12533* | D.C., Washington | MN702204 | - | MN702026 | - | MN702091 | MN702275 | MN702318 | MN702126 | MN702162 | MN702360 |
| *V. cinerea* var. *floridana* | *Wen 12552* | D.C., Washington | MN702209 | MN702241 | MN702031 | - | MN702096 | MN702280 | MN702323 | MN702131 | MN702167 | MN702365 |
| *V. flexuosa* var. *flexuosa* | *Wen 12518* | Japan, Tokyo | MN702201 | - | MN702023 | - | MN702089 | MN702272 | MN702315 | - | MN702159 | MN702357 |
| *V. flexuosa* var. *parvifolia* | *Wen 12461* | China, Hubei Prov. | MN702199 | - | MN702021 | - | MN702087 | MN702270 | MN702313 | - | MN702157 | MN702355 |
| *V. labrusca* | *Herron 1* | Tennessee, Johnson Co. | MN702191 | MN702234 | MN702013 | MN702056 | MN702080 | MN702266 | MN702305 | MN702119 | MN702153 | MN702347 |
| *V. labrusca* | *Wen 12541* | Virginia, Fairfax Co. | MN702208 | MN702240 | MN702030 | - | MN702095 | MN702279 | MN702322 | MN702130 | MN702166 | MN702364 |
| *V. labrusca* | *Wen 12559* | New Jersey, Middlesex Co. | MN702211 | MN702243 | MN702033 | MN702058 | MN702098 | MN702282 | MN702325 | MN702133 | MN702169 | MN702367 |
| *V. labrusca* | *Wen 12571* | Massachusetts, Middlesex Co. | MN702215 | MN702247 | MN702037 | MN702062 | MN702102 | MN702286 | MN702329 | MN702136 | MN702173 | MN702371 |
| *V. labrusca* | *Wen 12574* | Connecticut, Fairfield Co. | MN702217 | MN702249 | MN702039 | MN702064 | MN702104 | MN702288 | MN702330 | MN702138 | MN702175 | MN702373 |
| *V. labruscana* “Concord” | *Wen 12529* | Virginia, Fairfax Co. | MN702202 | - | MN702024 | - | MN702090 | MN702273 | MN702316 | MN702124 | MN702160 | MN702358 |
| *V. labruscana* “Concord” | *Wen 12568* | Massachusetts, Middlesex Co. | MN702212 | MN702244 | MN702034 | MN702059 | MN702099 | MN702283 | MN702326 | MN702134 | MN702170 | MN702368 |
| *V. labruscana* “Concord” | *Wen 12570* | Massachusetts, Middlesex Co. | MN702214 | MN702246 | MN702036 | MN702061 | MN702101 | MN702285 | MN702328 | MN702135 | MN702172 | MN702370 |
| *V. lanata* | *Wen 12738* | California, cultivated | MN702230 | MN702262 | MN702052 | MN702076 | MN702115 | MN702301 | MN702344 | MN702150 | MN702188 | MN702382 |
| *V. mustangensis* | *Wen 11991* | Texas, San Antonio | MN702195 | - | MN702017 | - | MN702083 | MN702268 | MN702307 | - | - | MN702349 |
| *V. mustangensis* | *Wen 11998* | Texas, Comal Co. | - | - | - | - | - | - | MN702308 | - | - | MN702350 |
| *V. riparia* | *Wen 12013A* | Louisiana, Tangipahoa Parish | - | - | - | - | - | - | MN702310 | MN702122 | MN702156 | MN702352 |
| *V. riparia* | *Wen 12534* | Maryland, Montgomery Co. | MN702205 | - | MN702027 | - | MN702092 | MN702276 | MN702319 | MN702127 | MN702163 | MN702361 |
| *V. riparia* | *Wen 12565-2* | Massachusetts, Middlesex Co. | MN702233 | MN702265 | MN702055 | MN702079 | MN702118 | MN702304 | - | - | - | - |
| *V. riparia* | *Wen 12569* | Massachusetts, Middlesex Co. | MN702213 | MN702245 | MN702035 | MN702060 | MN702100 | MN702284 | MN702327 | - | MN702171 | MN702369 |
| *V. riparia* | *Wen 12572* | Connecticut, Fairfield Co. | MN702216 | MN702248 | MN702038 | MN702063 | MN702103 | MN702287 | - | MN702137 | MN702174 | MN702372 |
| *V. riparia* | *Wen 12576* | New York, Orange Co. | MN702219 | MN702251 | MN702041 | MN702066 | MN702106 | MN702290 | MN702332 | MN702140 | MN702177 | - |
| *V. riparia* | *Wen 12604* | Virginia, Wythe Co. | MN702226 | MN702258 | MN702048 | MN702072 | MN702111 | MN702297 | MN702340 | MN702146 | MN702185 | MN702379 |
| *V. rotundifolia* | *Wen 12610* | Virginia, Grayson Co. | MN702227 | MN702259 | MN702049 | MN702073 | MN702112 | MN702298 | MN702341 | MN702147 | - | MN702380 |
| *V. thunbergii* | *Wen 12507* | Japan, Tokyo | MN702200 | - | MN702022 | - | MN702088 | MN702271 | MN702314 | MN702123 | MN702158 | MN702356 |
| *V. vinifera “Pinot Noir”* | *Wen 12582* | Virginia, Rockbridge Co. | MN702221 | MN702253 | MN702043 | MN702068 | MN702108 | MN702292 | MN702335 | MN702143 | MN702180 | MN702377 |
| *V. vinifera “Reichensteiner”* | *Wen 12746* | California, cultivated, DVIT 3107 | MN702232 | MN702264 | MN702054 | MN702078 | MN702117 | MN702303 | MN702346 | MN702152 | MN702190 | MN702384 |
| *V. vinifera “Riesling”* | *Wen 12581* | Virginia, Rockbridge Co. | MN702220 | MN702252 | MN702042 | MN702067 | MN702107 | MN702291 | MN702334 | MN702142 | MN702179 | MN702376 |
| *V. vinifera “Syrian”* | *Wen 12734* | California, cultivated, DVIT 2162; VIVC number 8467, donated to the National Clonal Germplasm Repository at UC Davis by a member of the California Rare Fruit Growers. | MN702229 | MN702261 | MN702051 | MN702075 | MN702114 | MN702300 | MN702343 | MN702149 | MN702187 | MN702381 |
| *V. vinifera “Thompson Seedless”* | *Wen 12580* | Virginia, cultivated | - | - | - | - | - | - | MN702333 | MN702141 | MN702178 | MN702375 |
| *V. vinifera* subsp. *sylvestris* | *Wen 12742* | California, cultivated, originally from Tunisia, likely cultivar “Tebaba” | MN702231 | MN702263 | MN702053 | MN702077 | MN702116 | MN702302 | MN702345 | MN702151 | MN702189 | MN702383 |
| *V. vulpina* | *S. Lutz 2014* | Maryland, Fredrick Co. | MN702192 | - | MN702014 | - | MN702081 | MN702267 | MN702306 | MN702120 | MN702154 | MN702348 |
| *V. vulpina* | *Wen 12006* | Texas, Montgomery Co. | MN702197 | MN702237 | MN702019 | - | MN702085 | - | - | - | - | - |
| *V. vulpina* | *Wen 12023* | Tennessee, McMinn Co. | - | - | - | - | - | - | MN702312 | - | - | MN702354 |
| *V. vulpina* | *Wen 12530* | Virginia, Fairfax Co. | MN702203 | - | MN702025 | - | - | MN702274 | MN702317 | MN702125 | MN702161 | MN702359 |
| *V. vulpina* | *Wen 12596* | Virginia, Dickenson Co. | MN702224 | MN702256 | MN702046 | MN702071 | MN702110 | MN702295 | MN702338 | MN702145 | MN702183 | MN702378 |
